# Supplementary material for: Complexome profiling of the Chlamydomonas psb28 mutant reveals TEF5 as an early PSII assembly factor
Source: Plant Cell. 2025 Mar 17;37(6):koaf055. doi: 10.1093/plcell/koaf055 (PMC12164590; doi:10.1093/plcell/koaf055)
Supplement: koaf055_Supplementary_Data [file koaf055_supplementary_data.zip › psb28_interactive.zip!/plots/intensity/qualityPlots/peptides/CGL78_Cre12.g490500.t1.2.html]

### Cre12.g490500.t1.2

Trivial name: CGL78  
SeqCoverage [median]: 30.90  
Mass: 23.29 kDa  
  
 

| Sequence | MissedCleavage | Mass [Da] | wtIntensity | muIntensity | Score |
| --- | --- | --- | --- | --- | --- |
| AAAPAPAAASADK | 0 | 1110.57 | 19936.93 | 3920.52 | 78.56 |
| DFFIVPNPK | 0 | 1075.57 | 41956.43 | 5714.32 | 125.25 |
| EQNKERDFFIVPNPK | 2 | 1859.95 | 345.30 | 0.00 | 28.28 |
| ERDFFIVPNPK | 1 | 1360.71 | 2137.01 | 1897.43 | 59.54 |
| FFKEQNK | 1 | 939.48 | 0.00 | 339.81 | 127.12 |
| FFKEQNKER | 2 | 1224.63 | 3299.30 | 860.98 | 109.66 |
| FPEQAK | 0 | 718.36 | 0.00 | 676.55 | 92.69 |
| SMPASEVLAAGEALPDFKPDGK | 1 | 2229.10 | 50466.72 | 40693.29 | 159.03 |
| WLDAKFPEQAK | 1 | 1331.69 | 4098.86 | 533.38 | 78.86 |
|  |  |  | 122240.5 | 54636.3 |  |
